# South West Vascular Surgeons

**Published:** 1992-08

**Authors:** 


### Abstracts of South West Vascular Surgeons

### Meeting held at St. Mary's Hospital, Portsmouth, 14 February 1992

### PSEUDOXANTHOMA ELASTICUM CAUSING CLAUDICATION.

W. G. Prout.
Queen Alexandra Hospital, Portsmouth.

Pseudoxanthoma elasticum is a heritable disorder of elastic fibre leading to characteristic skin changes, angioid occular streaking and cardio-vascular problems.

A 34 year old man presenting with intermittent claudication is presented.

The clinical presentation and pathogenesis of this rare condition are discussed.

### CHANGES IN MIDDLE CEREBRAL ARTERY VELOCITY WITH CAROTID CLAMPING: CORRELATION WITH STUMP PRESSURE.

T. R. Magee. A. H. Davies. J. Hayward, R. N. Baird, M. Horrocks
Vascular Studies Unit, Bristol Royal Infirmary.

The value of shunting and stump pressure measurement is controversial.

Thirty six consecutive carotid endarterectomies have been studied prospectively. Middle cerebral artery velocity was monitored intraoperatively in all patients and internal carotid artery stump pressure was measured at the time of clamping.

| | | |
|---|---|---|
| **Stump pressure** (mmHg) | 64.7 | (47.6-81.7)* |
| **% Stump pressure** (% of systemic) | 45.7 | (34.0-57.4)* |
| **Vmca** (change on clamping, cm/s) | 14.9 | (5.6-24.2)* |

The stump pressure measurements, whether expressed as an absolute or a percentage, did not correlate well with the change in Vmca on clamping ($r = 0.359$ and $r = 0.258$).

As judged by transcranial Doppler, stump pressure measurement is a poor indicator of cerebral perfusion during carotid clamping.

* = 95% confidence intervals.

### SPIRITUAL HEALING IN THE CONSERVATIVE MANAGEMENT OF VENOUS ULCERATION

A. D. R. Northeast, and K. G. Burnand
Surgical Unit, St. Thomas' Hospital, London.

We compared the time to total healing of ulcers of proven venous aetiology, in a randomised single blind trial of compression bandaging with or without spiritual healing. Ischaemia, rheumatoid and sickle disease were excluded. Patients were managed with paste, Tensopress, and tubular bandages. The treated patients were then seen elsewhere by a Confederation of Healing member.

| | n | Ulcer area cm$^2$ | Healing Time weeks | 3/12 healed | 12/12 healed |
|---|---|---|---|---|---|
| Control | 28 | 14.6 (1-82) | 9 (2-33) | 76% | 100% |
| Treated | 34 | 12.8 (1-62) | 11 (4-63) | 54% | 97% |
| Total | 62 | 13.6 | 10 (2-63) | 64% | 98% |

Kaplan-Meier survival analysis shows no significant difference in healing rate between groups, suggesting that spiritual healing has no influence. One ulcer remained unhealed at 12 months showing that most true venous ulcers may be healed by assiduous compression bandaging.

### FINANCIAL AND CLINICAL BENEFITS OF NON-INVASIVE ASSESSMENT OF THE LOWER LIMB ARTERIES FOR ANGIOPLASTY

A. H. Davies. T. R. Magee, S. E. A.Cole, J. K. Hayward, R. Parry*, P. Murphy*, R. N. Baird, M. Horrocks.
Departments of Vascular Studies & Radiology*,
Bristol Royal Infirmary.

Noninvasive assessment of the lower limb vasculature may avoid unnecessary arteriography in claudicants.

Colour Duplex scanning of femoral and popliteal arteries was performed and the pulse generated run-off (PGR) system was used to assess the distal vasculature. In 65 lower limbs Duplex gave the following results compared to angiography. Twenty three lesions were correctly identified as being suitable for angioplasty.

The cost of diagnostic angiography was £330 compared to £52 for non-invasive assessment. By using this method a potential saving of £11,676 could have been made.

A combination of Duplex scanning and PGR offers a noninvasive and cost effective alternative to diagnostic angiography for clinically suspected infra-inguinal arterial disease presenting as claudication.

### AORTIC DILATATION AMONGST RELATIVES

D. C. R. Adams, B. R. Tulloh, K. R. Poskitt
Cheltenham General Hospital.

Aortic aneurysmal disease may have a genetic basis for inheritance. We have studied the prevalence of aortic enlargement amongst first degree relatives of aneurysm patients to assess the suitability for screening.

First degree relatives of 100 consecutive abdominal aortic aneurysm patients were contacted. One hundred and four relatives (>50 yrs) were traced. Two (2%) had known aortic aneurysms. A further 76 (73%) proceeded to ultrasound scanning. In total, 9 of 39 males (23%) and 2 of 39 females (5%) were found to have enlarged aortas (AP diameter > 2.5cm).

In conclusion we feel that male first degree relatives are an appropriate sub population for aortic aneurysm screening.

### QUALITY OF LIFE AFTER GRAFTING FOR AORTIC ANEURYSM.

T. R. Magee, D. J. A. Scott, J. A. St. Johnston,
W. B. Campbell, R. N. Baird, M. Horrocks.
Bristol Royal Infirmary and Royal Devon and Exeter Hospital.

This study examined quality of life a year or more after aortic grafting in 211 consecutive patients (186 male) aged 48-87 (median 74 years). There were 124 elective and 77 ruptured aneurym procedures. By the time of this review 165 patients were still alive, and 131 were interviewed (86 elective and 45 ruptured.

The mental and physical state of patients was assessed using a Rosser index to calculate an average quality of life (QoL) score. Those who had had elective aneurysm operations had similar QoL scores after operation as before, while scores were lower postoperatively in the ruptured aneurysm group.

The commonest specific complaint was decreased sexual potency in men, affecting 38 of 119 (32%) with initially normal sex function.

### A COMPARATIVE AUDIT OF VASCULAR SURGICAL PRACTICE AT A DISTRICT GENERAL AND TEACHING HOSPITAL

J. A. Michaels, D. Browse, R. B. Galland, P. Lamont, D. Gray, J. Collin, P. J. Morris.
John Radcliffe Hospital, Oxford and Royal Berkshire Hospital, Reading.

A three month audit compared the vascular surgical practice at a Teaching Hospital (TH) and a neighbouring District General Hospital (DGH) serving similar populations. The source and nature of referral, severity of presenting symptoms and initial management were recorded. The DGH received 128 new referrals and the TH received 153 with a similar mix of diagnoses (see table, referrals from out of area shown in brackets).

| | Critical ischaemia | Claud-ication | Leg pain | AAA | Acute ischaemia | Carotid neck etc. | Other | Total |
|---|---|---|---|---|---|---|---|---|
| DGH | 45 (1) | 33 | 10 | 16 | 8 | 1 | 15 (1) | 128 (2) |
| TH | 53 (3) | 31 (4) | 14 (1) | 15 (0) | 7 (2) | 14 (3) | 19 (2) | 153(15) |

Of those with critical ischaemia the TH had more emergency referrals (59% vs 38%) and more with ulceration or gangrene (74% vs 44%). Severity of claudication was similar with just over half claudicating at less than 200 yds (58% TH vs 52% DGH). More patients with carotid disease or thoracic outlet syndrome were referred to the TH. Referrals from outside the direct catchment area were greater for TH (9.8% vs 1.6%). Both hospitals have similar investigation facilities but out-patient iv DSA is used more at the DGH and in-patient ia DSA at the TH. Over the same period 51 major vascular operations were carried out at the DGH and 84 at the TH, where there were more carotid endarterectomies (9 vs 0), distal bypasses (9 vs 0), re-explorations (10 vs 3) and a higher rate of vein usage for femoro-popliteal grafts (87% vs 41%). These results suggest that the more specialist vascular service available at the TH is provided largely to its local population.

### COMPUTERIZED AUDIT FOR VASCULAR SURGEONS

J. J. Earnshaw, J. K. Hayward, M. Horrocks, R. N. Baird.
Vascular Studies Unit, Bristol Royal Infirmary.

This audit of vascular surgery in a teaching hospital was facilitated by computerized data collection. A total of 2,075 patients had 2,628 procedures over the six year period 1985-1990.

Vascular workload increased by 50% over this period. Mean hospital stay is twice as long for vascular patients as general surgery patients (14 days vs 6 days).

The overall mortality was 10.4% ranging from 1.7% to 31.9%. Early re-operation for the complications of vascular reconstruction was required in 9.1%.

Vascular surgery is expensive, time consuming and expanding. Audit is a pre-requisite for surgeons wishing to attract and maintain a vascular practice.

### COMPLICATIONS OF ARTERIAL GRAFTING IN EXETER 1987-91

L. Tambeur, V. Geens, W. B. Campbell.
Royal Devon and Exeter Hospital.

During this five year period 501 bypass grafts to the lower limbs were implanted. There were 149 aortoiliac, 165 femoropopliteal, 78 femorotibial, 65 extra-anatomic, and 44 miscellaneous grafts.

Postoperative bleeding occurred early in 15 (3.0%), and later because of infection in 3 (0.6%). Early graft occlusion was seen in 2.9% aortofemoral, 10.3% femoropopliteal, and 24.0% femorotibial grafts. There was only 1 case of distal embolism after aortic surgery.

Wound dehiscence and infection were commonest after femorotibial bypass (13.5%). Weeping of lymph or serous fluid was also commonest after femorodistal grafts - 4% for vein and 1.3% for PTFE. Lymphatic collections occurred after 1.2% operations involving groin incisions.

Graft sepsis was usually late, affecting 6% aortofemoral, 2.8% femorodistal, and 7.7% extra-anatomic grafts. No intra-abdominal aortic grafts became infected.

The overall incidence of amputation after grafting was 6.8%.

### FALSE LEFT BRACHIAL ARTERY ANEURYSM FOLLOWING HUMERAL FRACTURE

C. S. Perkins.
Queen Alexandra Hospital, Portsmouth.

An eighty-four year old female fractured her left humerous in a fall in 1991. In June 1991 she had one episode of ischaemia of the left hand treated with Heparin. In September 1991 further left hand ischaemia was treated with Heparin and in November 1991 further ischaemia and swelling of the left hand was associated with a large pulsating mass in the left shoulder. Exploration and radiology of the left axillary artery confirmed an aneurysm of the brachial artery. Following clot evacuation the bleeding was controlled proximally by a clamp and distally by a balloon catheter. The arteriotomy was closed 60 proline and the fracture edge excised for 2 cm. Following the repair of the artery the hand remained warm although the pressures were low. Function was normal. This case illustrates the late presentation of a false aneurysm but the warning signs of ischaemia were not investigated.

### AORTIC ANEURYSM REPAIR WITHOUT HOMOLOGOUS BLOOD TRANSFUSION

B. R. Tulloh, C. P. Brakespear, S. C. Bates, D. C. R. Adams, R. G. Dalton, M. A. Durkin, J. B. Bristol & K. R. Poskitt.
Cheltenham General Hospital.

Blood replacement in abdominal aortic aneurysm (AAA) repair places demands on blood bank resources and exposes patients to the risks of transfusion. To assess the feasibility of elective AAA repair without using homologous transfusion, ten consecutive patients each donated two units of blood over the two weeks preoperatively and had intraoperative blood loss salvaged with the Haemonetics Cell-Saver 111- plus.

The first two patients were mistakenly transfused in breach of the study protocol but none of the remaining patients has required homologous transfusion. We conclude that elective AAA repair can be safely performed without using homologous blood.

### MAJOR AMPUTATION COMPLICATING INFLAMMATORY BOWEL DISEASE

P. W. Leopold, M. R. Thompson.
St. Mary's Hospital, Portsmouth.

In the absence of significant arteriosclerosis or cardiac disease limb loss is a rarity. Other causes including trauma, neoplasia congenital vascular and primary arterial wall abnormalities make up the remainder.

Major limb loss complicating inflammatory bowel disease (IBD) is an extreme rarity in the absence of the above. We present 2 patients aged 27 and 60 years, with IBD complicated by acute limb ischaemia resulting in forearm amputations and a review of the literature.

### AMPUTATION IN EXETER 1987 - 91

W. B. Campbell, E. A. Rutter,
J. A. St. Johnston, V. F. M. Kernick.
Royal Devon and Exeter Hospital.

During this 5 year period 212 patients underwent 230 primary major lower limb amputations. Eighteen lost both legs, and a further 13 became bilateral amputees (15% overall). Diabetics comprised 55% of the bilateral amputees, but only 33% of the total patient group.

Primary amputation was below knee in 149 65%), Gritti Stokes in 8 (3%) and above knee in 73 (32%). Revision was required in 43 (19% cases) - to a higher level in 29 below knees (19%) and 1 Gritti Stokes. Overall mortality was 14%.

Amputation was preceded by attempted limb salvage by arterial grafting in 69 cases (36 within 30 days, and 13 after more than a year). Fifty seven percent of these were below knee and the revision rate was 13%.

Of patients alive at one month 66% were referred for limb fitting. Sixty nine patients fitted with prostheses were evaluated more than six months after amputation, and 52% were able to walk beyond their house and garden.

### SMALL SCALE COMPUTER DECISION SUPPORT SYSTEMS FOR VASCULAR SURGERY

C. J. Ranaboldo, A. B. D. Chant, J. Davies and P. Soper.
Royal South Hampshire Hospital, Southampton.

The clinical decisions made in vascular surgery are complex. A need exists to relate a specific case to both specific local experiences and to the world literature.

We have implemented an information technology system that mirrors the case notes. It relates each patient's details to a separate knowledge base that contains key information distilled from the world literature.

The representation of the existing clinical material is accurate. The computer provides appropriate advice about the management of most patients and relates its knowledge of each case with reasonable efficiency to the literature. It is now undergoing prospective evaluation.

### IMMEDIATE EFFICACY OF ENDOSCOPIC THORACIC SYMPATHECTOMY ASSESSED BY PEROPERATIVE PALMAR TEMPERATURE MEASUREMENT

J. F. Chester.
Taunton and Somerset Hospital.

Endoscopic transthoracic sympathectomy was first described in 1978, and is now regarded as the treatment of choice for palmar hyperhidrosis. Most patients report dry hands immediately on return from theatre, and about 80% are very much improved thereafter.

Using a disposable temperature probe (Mallinckrodt Hi-ho skin temperature sensor, Mallinckrodt UK Ltd, Northampton) affixed to the patient's palm on the relevant side during the procedure, palmar temperature rises of between 1.1 and 2.6 degrees centigrade (average 1.6 degrees C) have been recorded within 3 minutes of coagulation of the sympathetic chain in six cases ($p<0.03$; Wilcoxon matched pairs test). With this technique, we have found that the procedure can be completed secure in the knowledge that sympathectomy has been achieved, and in each case, palmar temperature measurement has proved to be an inexpensive teaching aid in the demonstration of living physiology.

### DUPLEX SCANNING FROM SCRATCH

M. G. Wyatt, P. G. Niblett, D. H. Bennett, S. L. Phillips, D. James, W. B. Campbell.
Departments of Surgery and Clinical Measurement.
Royal Devon and Exeter Hospital (Wonford), Exeter.

Since 1986, 446 patients have undergone 910 carotid duplex scans. Three quarters of patients described focal symptoms such as transient ischaemia (162), amaurosis fugax (118), or completed stroke (64). Seventy five percent of arteries were normal (682). 205 stenoses were detected and 23 arteries were occluded. Ninety one arteries were also examined with arteriography, with agreement in 86% of cases. Duplex missed 4 stenoses, but none were severe (>70%). Two occlusions were missed and 3 severely stenosed arteries were reported as occluded.

These scans have resulted in a total of 44 carotid endarterectomies being performed, 20 on duplex findings alone and 24 after arteriographic confirmation. The majority of arteriograms were performed in the early days, but as confidence in the technique has increased, a greater proportion of surgery is now performed on duplex findings alone.

